# miR-146a-5p and miR-193a-5p Synergistically Inhibited the Proliferation of Human Colorectal Cancer Cells (HT-29 cell line) through ERK Signaling Pathway

**DOI:** 10.34172/apb.2021.085

**Published:** 2020-09-08

**Authors:** Saeed Noorolyai, Elham Baghbani, Dariush Shanehbandi, Vahid Khaze Shahgoli, Amir Baghbanzadeh Kojabad, Behzad Mansoori, Khalil Hajiasgharzadeh, Ahad Mokhtarzadeh, Behzad Baradaran

**Affiliations:** ^1^Immunology Research Center, Tabriz University of Medical Sciences, Tabriz, Iran.; ^2^Student Research Committee, Tabriz University of Medical Sciences, Tabriz, Iran.; ^3^Pharmaceutical Analysis Research Center,Tabriz University of Medical Sciences, Tabriz, Iran.

**Keywords:** miRNA-146a-5p, miRNA-193a-5p, Colorectal cancer, ERK pathway, Cell growth

## Abstract

***Purpose:*** The expression of miR-146a-5p and miR-193a-5p in colorectal cancer (CRC) is associated with cancer development, metastasis, and reduced survival rate of the tumor-suffered subjects. This examination aimed to assess the impact of these microRNAs (miRNAs) in CRC and their mechanisms in the proliferation and migration of cancer cells.

***Methods:*** miR-146a-5p and -193a-5p were transfected into the HT-29 cell line and assessed their impact on metastasis-related genes. The synergistic effects of these miRNAs on migration were evaluated by wound healing approach. To assess the influence of these miRNAs on the proliferation of and apoptosis of cells, the MTT test, annexin V staining test, and DAPI staining test were done. Then, the protein expression of extracellular-signal-regulated kinase (ERK) and phosphorylated ERK (p-ERK) were investigated.

***Results:*** miR-146a-5p and-193a-5p could inhibit the CRC cells proliferation, and could synergistically induce apoptosis in CRC cells, and also repressed cell migration, and could reduce p-ERK expression.

***Conclusion:*** miR-146a-5p and-193a-5p have an important role in cell viability and proliferation via ERK signaling pathway. Thus, the simultaneous use of these miRNAs may be suggested as a probable therapeutic strategy in this cancer therapy.

## Introduction

Colorectal cancer (CRC) is among the widespread kinds of cancers and is the third most ordinary cancer in the universe, with over 1.2 million incidences every year,^1^ the second in males and the third in females leading reason of cancer death, accounting for ~9% of all cancer deaths worldwide.^2,3^ Treatment approaches for CRC and metastatic CRC patients have experienced exhibitive alterations in the last decade and even though improved patient results, there still be fields for continuous expansion. In this topic, targeted therapy has a special place in new cancer therapy options, in which the identification of specific molecular targets is the most essential action.^1^ Nowadays, the role of a new group of non-coding RNAs called microRNAs (miRNA; miR) has been revealed in tumorigeneses.^4^ miRNAs play a crucial role in CRC biology, tumor progression, angiogenesis, carcinogenesis, invasion and metastasis.^5^ The introduction of miRNAs in recent years, as the chief regulators of genes, has created new hope in the treatment of cancer.^6^ The expression of some miRNAs in cancer is increased and acts as an oncogene, and some miRNAs are reduced in cancers and act as tumor suppressor (TS) miRNA, so these molecules are classified as oncogenes and TS miRNAs.^7^ Several studies have displayed that, among the TS miRNAs, the low expression of miR-146 a-5p and miR-193a-5p are connected with tumor development, metastasis, chemotherapeutic resistance, and poor survival rate of the cancer patients.^8,9^ Both of these miRNAs has tumor suppressor effects in CRC and other tumors, miR-146 a-5p has a crucial role in the inhibition of migration and proliferation in CRC.^8^ and also, the miR‐193a‐5p has especial biological roles in the pathogenesis of CRC such as cell growth, metastasis, and apoptosis.^9^ Considering fundamental molecular contrivances in CRC development and progression affords novel visions in developing new options for CRC treatments.^10^ Accumulative confirmations demonstrate that multiple signaling cascades have a fundamental role in the growth and expansion of CRC.^11^ Targeting these signaling pathways might be valuable for the treatment of CRC and may introduce new methods for the treatment of CRC and prepare it’s competent controlling in contrast with the formal therapy. Though, the expression of miR-146a-5p/-193a-5p effects multiple gene expression and signaling pathways, in CRC. The goal of the current investigation was to transfect these miRNAs to the CRC cell line and investigate the synergistic influences of this simultaneous replacement on underlying signaling pathways.

## Materials and Methods

### Cell culture

Human CRC cell lines, such as HT-29, HCT-116, and SW-480 were received from Pasture Institute (Tehran, Iran). The mentioned cell lines were cultured in RPMI‐1640 medium contained penicillin/streptomycin mixtures, which enriched by 10% fetal bovine serum (FBS) (Gibco, Maryland). Cultured cells were kept at 37°C with 5% CO_2_ and 95% humidified atmosphere, according to our previous study.^12^ HT-29 cell line was selected to continue the study because it had the lowest expression of both miR193a-5p and -146a-5p between these three cell lines.

### microRNA transfection

Negative control FITC-conjugated miRNA (NC‐miRNA) and miR-146a-5p/ -193a-5p sequences were designed and then acquired from Microcynth (AG, Switzerland). 2 × 10^5^ HT-29 cells were cultured in a 6-well-plate and kept for 24 hours. After removal of the medium, an antibiotics-free Opti-MEM medium, which supplemented with FBS was added (Gibco, Maryland). The FITC-conjugated miRNA and miRNA-146a-5p/-193a-5p sequences were transfected separately and simultaneously by jetPEI reagent (PolyPlus, France). After 24 hours, the transfection efficacy of FITC-conjugated miRNA was determined in HT-29 cells by flow cytometry (FCM) (MacsQuant Analyser 10, Miltenyi Biotech, Germany).

### RNA extraction and qRT‐PCR

Total RNA was extracted using TRIzol reagent from the cells (RiboEx). The concentration and the quality of isolated RNAs were measured by NanoDrop (Thermo Scientific, USA). The miRNA Reverse Transcription Kit (Exiqon, Denmark) was applied for cDNA synthesis in the assessment of miR-146a-5p/-193a-5p according to the Exiqon protocol. Also, the mRNA was reverse transcribed into cDNA with a kit (Biofact, South Korea). qRT‐PCR was done with the light cycler 96 instrument (Roche Diagnostics, Mannheim, Germany) with a Master Mix Kit (Biofact, South Korea). The primers sets were as described in Table 1. The assessment of miR-146a-5p/-193a-5p, C-Myc, ROCK, CXCR4, and E-cadherin were accomplished by qRT‐PCR. Finally, the relative expression ratio of the genes was normalized by U6 and GAPDH controls for miRNA and target genes, individually, by 2^-ΔΔCt^ method.

**Table 1:** Primer sequences in qRT-PCR

**Name of Genes**		**Sequences**
CXCR4	Forward	5′-TCTTCCTGCCCACCATCTACTC-3′
	Reverse	5′-TGCAGCCTGTACTTGTCCGTC-3′
E-cadherin	Forward	5′-TGCCCAGAAAATGAAAAAGG-3′
	Reverse	5′-GTGTATGTGGCAATGCGTTC-3′
C-Myc	Forward	5ˊ-AGGCTCTCCTTGCAGCTGCT-3ˊ
	Reverse	5ˊ-AAGTTCTCCTCCTCGTCGCA-3ˊ
ROCK	Forward	5ˊ-CTCCCTGTGTCAGACTGCTCTTT-3ˊ
	Reverse	5ˊ-GGCCTTGCAACCTTGGTCTCTTC-3ˊ
GAPDH	Forward	5ˊ-CAAGATCATCAGCAATGCCT-3ˊ
	Reverse	5ˊ-GCCATCACGCCACAGTTTCC-3ˊ
U6 (RNU6-1)	Forward	5′-CTTCGGCAGCACATATACTAAAATTGG-3′
	Reverse	5′-TCATCCTTGCGCAGGGG-3′
Hsa-miR-146a	Target sequence	5′-UGAGAACUGAAUUCCAUGGUU-3′
Hsa-miR-193a	Target sequence	5′-UCAUCUCGCCCGCAAAGACCC-3′

### Wound healing (scratch) assay

The wound healing approach was used for evaluation of the effects of each miR146a-5p and -193a-5p and their synergistic effects on the cellular migration. For this object, miR-193a-5p/ -146a-5p separately and combined were transfected into the cells, and then the photographs were taken from each well by an inverted microscope (Optika, Italy) from 0 until 48 hours following transfection. The movement of the treated cells from the border to the gap zone was estimated in transfected groups compared to controls.

### Cytotoxicity and combination effects of microRNAs transfection

For evaluation of cytotoxicity effects of miRNAs on CRC cell, MTT assay was assessed. In summary, 15 ×10^3^ HT-29 cells were cultured in a 96-well plate per well. After 75–80% confluency, the transfection of miRNAs was done separately and simultaneously manner. 24 and 48 hours following the transfection, 50 μL of prepared MTT solution (2 mg/mL in PBS) were incubated and kept at standard incubator conditions for 4 hours. Afterward, 200 μL DMSO was added to wells and kept for an additional 30 minutes at 37℃. Then, the optical density of the well was evaluated by a Sunrise ELISA reader (Tecan, Austria). All test samples were accomplished in triplicate. The analysis of the miR-146a-5p and -193a-5p was made to examine the interaction between these two miRNAs. The CI results, moreover identified as the combination index, in HT-29 cells was evaluated through the following formula: CI = [m1/ (m)1] + [m2/ (m)2] + [m1m2/(m)1(m)2]. m1 and m2 are the respective combination concentrations of miR-193a-5p/-146a-5p that produce an effect of 50% growth inhibition, with (m)1 and (m)2 being the corresponding single concentrations for miR-146a-5p and -193a-5p. As a consequence, there are three categories of CI such as CI < 1 (synergism), CI = 1 (summation), and CI > 1 (antagonism).

### Apoptosis assay

To evaluate apoptosis induction by miR-193a-5p/-146a-5p transfection, annexin-V, and propidium iodide (PI) double staining kit (Exbio, CZ) were applied. After transfection, cells were harvested from each well, treated with 500 μL of binding buffer, and after that with 5 μL of annexin‐V-FITC and 5 μL of PI for 15 minutes on ice in the dark place. The apoptosis rates of each group of cells were measured using previously mentioned FCM apparatus and data were measured by utilizing FlowJo software.

### DAPI staining

To confirm the apoptosis induction by miRNAs, we used DAPI staining in addition to FCM analysis. Briefly, 15×10^3^ HT-29 cells were seeded in 96 well plate. Following 48 hours transfection and then PBS washing (3×), 100 μL of Formalin 4% was added to each well and kept for 2-4 h at 37℃, to fix the cells. Then, after washing, the cells were permeabilized with 0.1% Triton X-100 for 15 min stained with DAPI for 10 minutes following PBS washing (3×). The fragmented chromatin of apoptotic cells was evaluated using a live imaging system with Cytation 5 (Biotek, USA).

### Western blotting

The whole protein was isolated by RIPA lysis buffer (Santa Cruz, CA) according to the recommended protocol. Afterward, 50 µg of every protein sample was separated by using SDS‐PAGE electrophoresis and blotting in the PVDF membrane (Roche, Basel, Switzerland).^13^ The PVDF was blocked in blocking buffer [0.5% (v/v) Tween 20 in PBS] for 2 hours on a shaker at room temperature (RT) and next treated with monoclonal antibodies against extracellular-signal-regulated kinase (ERK), p-ERK, and β‐actin as a reference protein overnight at 4℃ (1: 1,000; v/v). Next, the PVDF membranes were treated with HRP-conjugated secondary antibodies for ERK, p-ERK, and β‐actin (1: 3,000, v/v) for 2 hours at RT in the shaking situation. The protein bands were pictured with an electrochemiluminescence detection kit (Roche Diagnostics). The strength of protein bands was evaluated via ImageJ software and standardized with the β-actin band.

### Statistical analysis

All values are presented as mean ± SD. GraphPad Prism 6 software was applied for statistical analysis. One-way and two-way analyses of variance were done to demonstrate statistical differences among two or more groups, followed by Sidak and Tukey tests. Statistically significant differences were assessed at *P*  <  0.05.

## Results

### miR‐193a‐5p and -146a-5p were downregulated in CRC cell lines

**Figure 1:**
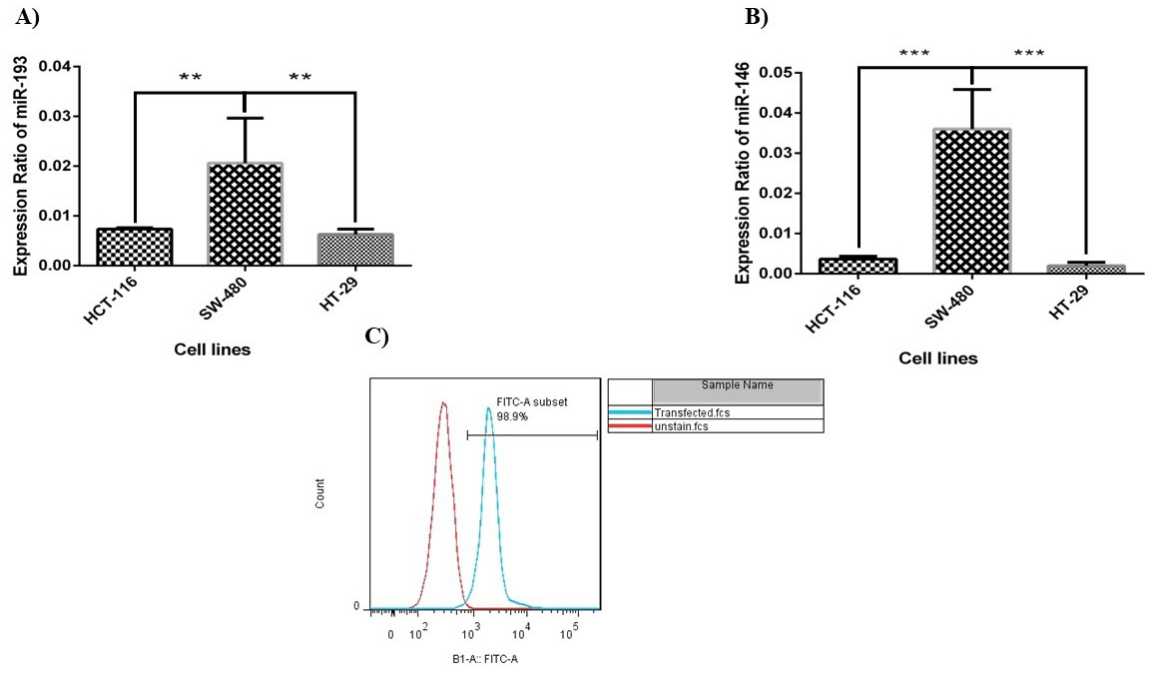
Relative expression levels of miR‐193a‐5p (A) and miR-146a-5p (B) in colorectal cancer cell lines evaluated with each other (**P  <  0.001). (C) Evaluation of transfection efficacy by flow cytometry. According to the results of flow cytometry, the transfection rate of HT-29 cells was 98.9%.

We evaluated miR‐193a‐5p/-146a-5p expression in different CRC cell lines (i.e. HCT-116, HT‐29, and SW-480) and the outcomes presented that the expression ratio of miR‐146a‐5p and-193a-5p was meaningfully low in all these cell lines. According to Figure 1, between these cell lines, HT-29 cells have the lowest expression level of miRNA-146a-5p/-193a-5p. Therefore, according to the results, the HT-29 cells were selected to continue the study. The HT-29 cells were significantly transfected with control FITC-conjugated miRNA. The efficiency of the cell transfection rate was evaluated by FCM, and the results demonstrated that the amount of transfection was about 98.9% (Figure 1C).

### miR‐146a‐5p and -193a-5p were upregulated following mimic transfection in HT‐29 cells

**Figure 2:**
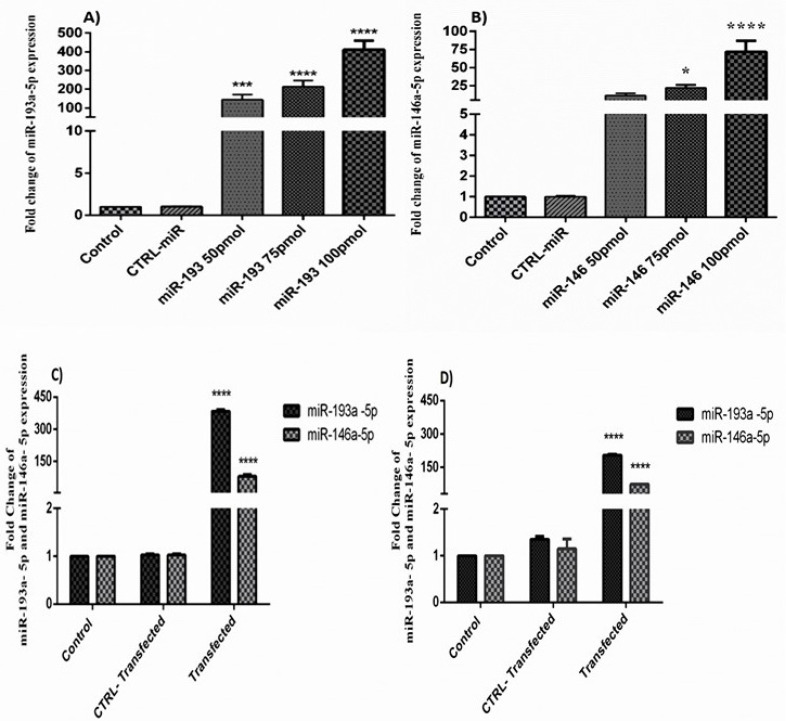
(A) The effect of miR-193a-5p replacement in the HT-29 cell line. The expression of miR-193a-5p was significantly increased in 100 pmol of miR-193a-5p (****P < 0.0001, *** *P* <  0.001). (B) The effect of the miR-146a-5p replacement in the HT-29 cell line. The expression of miR-146a-5p was significantly increased in 100 pmol of miR-146a-5p (****P < 0.0001, * *P* <  0.01). (C) The effect of the simultaneous replacement of miR-193a-5p and -146a-5p in the HT-29 cell line. The expression of miR-146a-5p and miR-193a-5p was significantly increased in comparison with control cells and control transfected cells in 24h after transfection (****P < 0.0001). (D) The effect of the co-treatment of miR-193a-5p and -146a-5p in the HT-29 cell line. The expression of miR-146a-5p and miR-193a-5p was significantly increased in comparison with control cells and control transfected cells in 48 h after transfection (****P < 0.0001).

The consequences of qRT-PCR displayed that transfection of miR-146a-5p and -146a-5p, enhanced the expression ratio of these miRNAs in HT-29 cells in a dose-dependent pattern. Besides, the expression of these miRNAs was significantly increased in the group of cells that transfected with miR-193a-5p/-146a-5p simultaneously (Figure 2).

### miR‐146a‐5p and -193a-5p replacement downregulated the expression of E-cadherin, ROCK, CXCR4, and c-Myc in HT-29 cells

**Figure 3:**
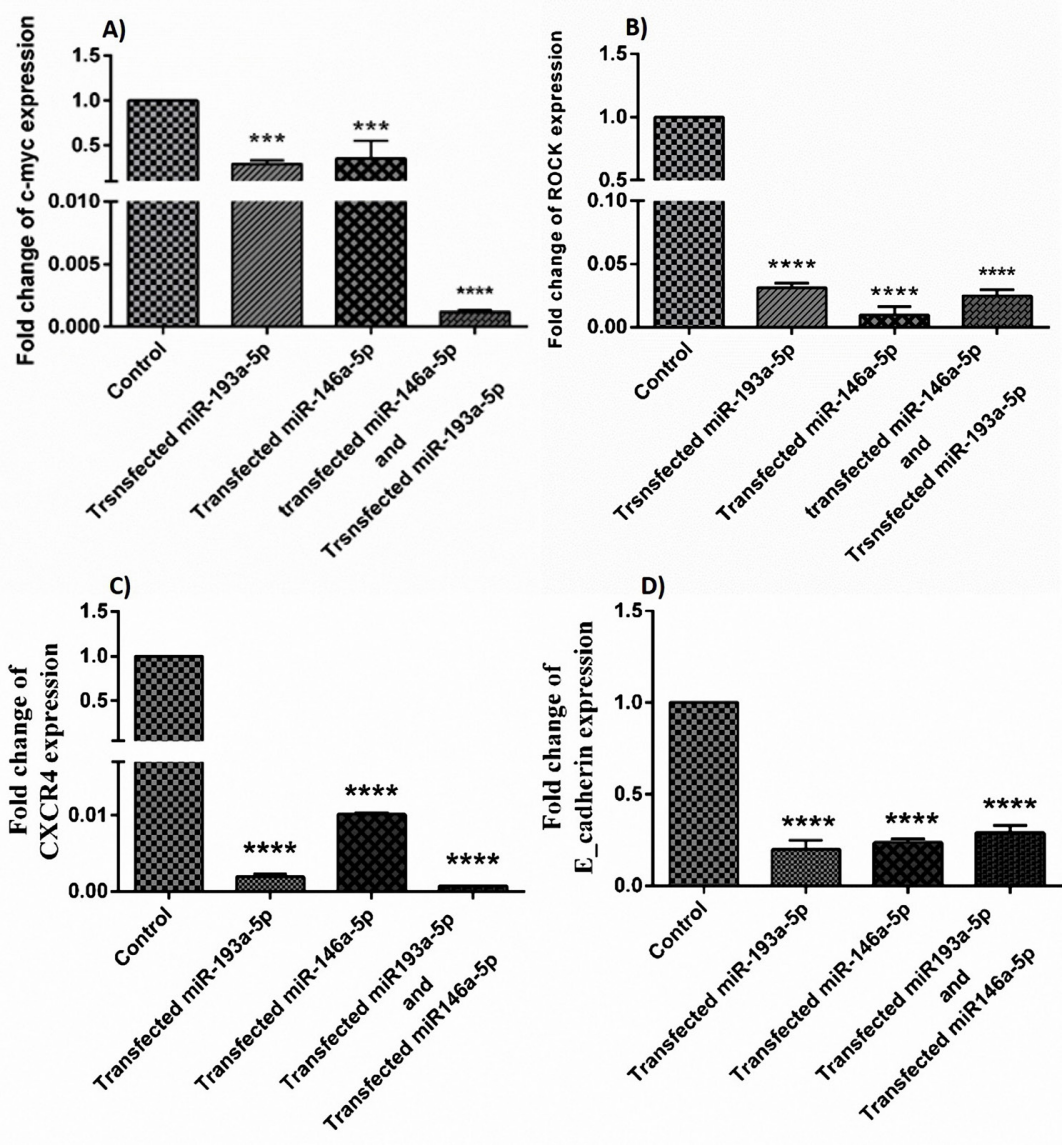
The effects of upregulation of miRNA-146a-5p and -193a-5p alone and in combination with each other on the expression of (A) C-Myc, (B) ROCK, (C) CXCR4, (D) E-cadherin in comparison with the control group (***P < 0.001, **** P < 0.0001).

The qRT-PCR results showed that the co-treatment and mono- treatment with miR-146a-5p/-193a-5p reduced the expression ratio of C-Myc (Figure 3A), ROCK (Figure 3B), CXCR4 (Figure 3C) and E-cadherin (Figure 3D) significantly. We checked the binding sites between miR-146a and miR-193a and these genes, according to the bioinformatics databases like NCBI, to the validation of these results.

### miRNA-146a-5p and -193a-5p individually and synergistically reduced the CRC cells migration rate

**Figure 4:**
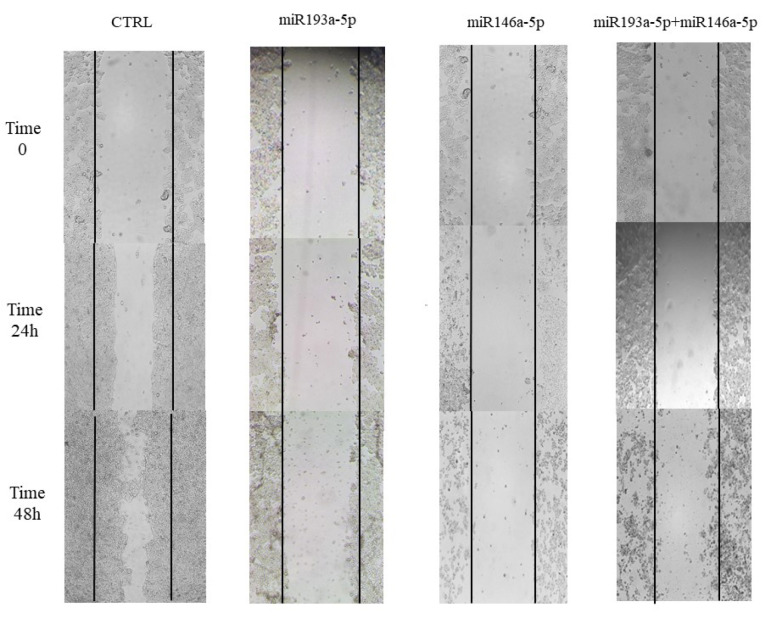
The effect of miR-146a-5p and -193a-5p replacement on HT-29 cell migration. The migration of HT‐29 cells in the wound zone was checking for 0, 24, and 48 h. Data displayed that migration was decreased in miR-193a-5p and -146a-5p transfected cells, alone and in combination with each other in HT-29 cells, in comparison with the control group.

As demonstrated in Figure 4, mimic transfection of miRNA-146a-5p/-193a-5p either alone or combined inhibited HT‐29 cells migration. This treatment synergistically had considerable inhibition of cell migration at 48 hours compared with control cells.

### miR-146a-5p and -193a-5p replacement reduced the HT-29 cells proliferation

**Figure 5:**
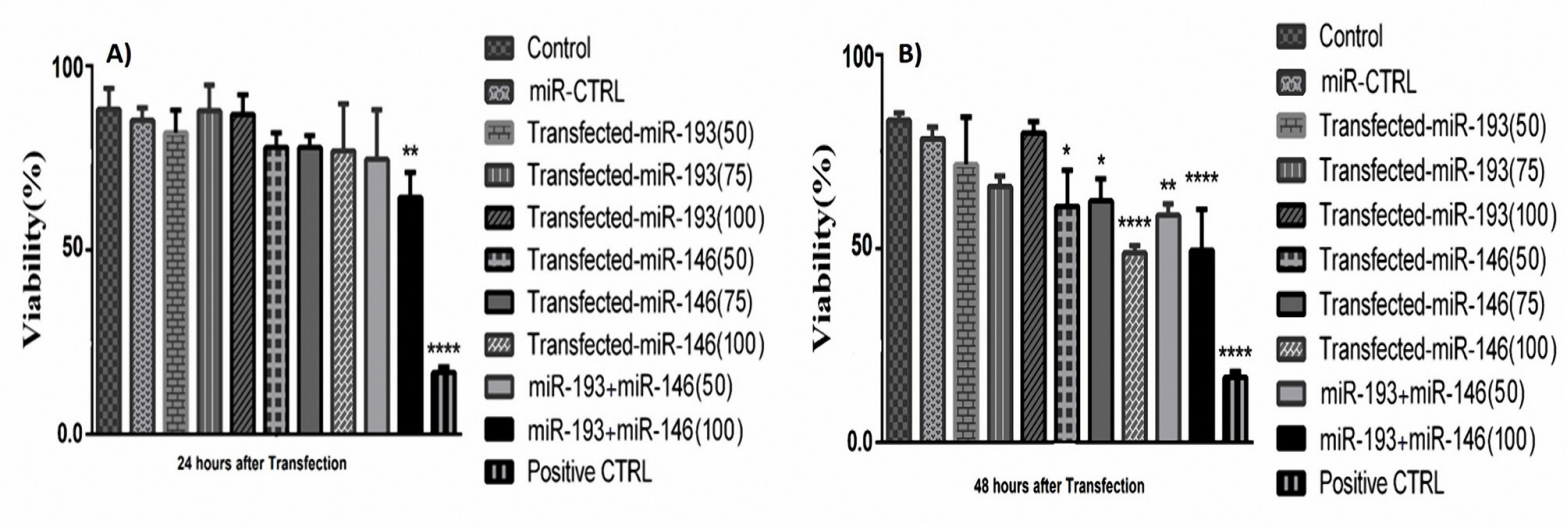
(A) The results of MTT assay 24 h after transfection with different doses of miR-146a and -193a. The survival ratio of cells that simultaneously transfected with both miRNAs was synergistically decreased (** *P* <  0.001). B) MTT test results after 48 h transfection with different doses of miRNAs. The survival rate was significantly reduced in groups that were transfected with miR-146a and the group of cells that simultaneously transfected with both miRNAs (****P < 0.0001, ** *P* <  0. 001, * *P* <  0. 01).

The results of the MTT test revealed that the cell survival ratio was reduced in the group of HT-29 cells with miR-146a/ -193a co-treatment compared to the control, 24h after transfection (Figure 5A). Within 48 h after transfection, in all three doses of mimic miRNA-146a, the survival rate was significantly decreased in a dose-dependent pattern. And, also in groups that simultaneously transfected with miR-146a and -193a, the survival rate was decreased synergistically in comparison with control and the group transfected with miR-control (Figure 5B). The combination index (CI) was calculated for evaluating the synergistically inhibitory impacts of miRNAs. The quantitative explanation for additive effect (CI = 1), synergism (CI less than 1), and antagonism (CI higher than 1) in miRNA combinations. Our data indicate CI < 1 indicates a meaningfully synergistic effect.

### Simultaneous treatment with miR-193a-5p and -146a-5p synergistically enhanced the apoptosis of CRC cells

**Figure 6:**
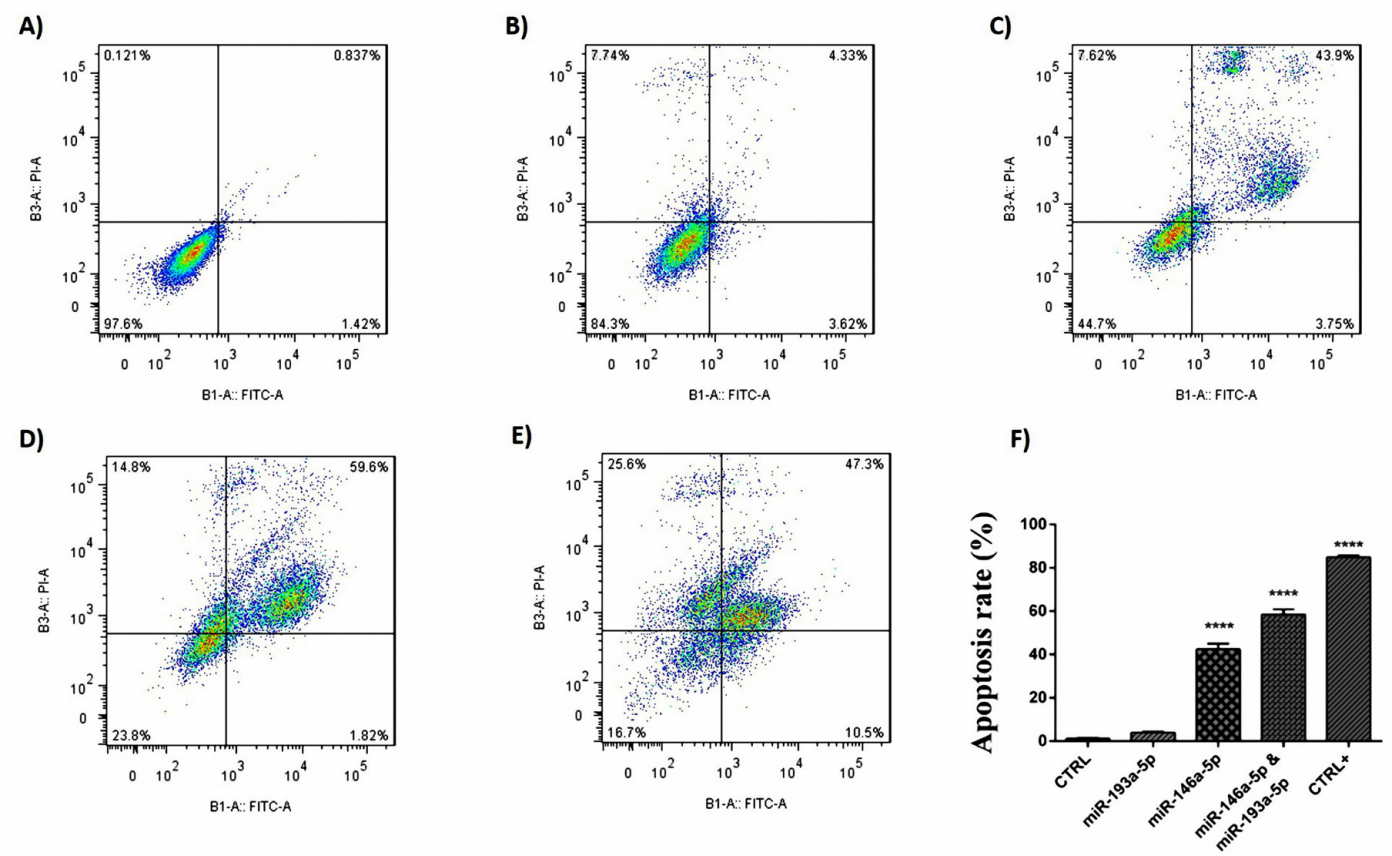
Flow cytometry test using annexin-V and PI staining method. Apoptosis was significantly observed in the group of cells that transfected with miRNA-146a-5p mimic and cells that co-transfected with both miRNAs, in comparison with the control group, (A) Control stain, (B) miR193a-5p, (C) miR146a-5p, (D) miR193a-5p and miR146a-5p, (E) Positive CTRL, and (F) Apoptosis rate (****P < 0.0001).

**Figure 7:**
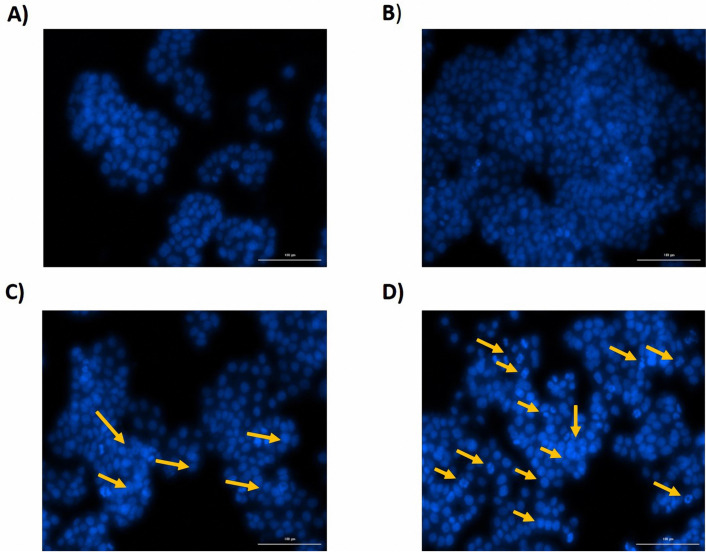
DAPI Staining. Apoptosis was meaningfully observed in the group of cells that transfected with miRNA-146a-5p mimic and also in the group of cells that simultaneously transfected with miR-146a-5p and -193a-5p, in comparison with the control group. The nucleus of the apoptotic cells is visible in the form of a blue sliced and fragmented, which is indicated by the arrows. (A) CTRL, B) miR193a-5p, C) miR-146a-5p, D) miR-146a and- 193a-5p).

According to the results of FCM assay, miRNA-146a-5p transfection and co-transfection of miRNA-146a/-193a-5p synergistically, induced the apoptosis of HT-29 cell line. On the other way, miRNA-193a-5p had no considerable impact on the apoptosis rate of the HT-29 cell line (Figure 6). According to the results of DAPI staining, it is consistent with the results of FCM test using PI and annexin V staining, in the group of cells that transfected with miRNA-146a-5p and in the group of cells that were co-transfected with miR-146a-5p/-193a-5p, apoptosis was detected compared with control groups. The nucleus of these cells was condensed and fragmented, and they were observable in blue color (Figure 7).

### Co-treatment with miR-146a-5p and -193a-5p synergistically inhibited the ERK signaling pathway in HT-29 cell line

**Figure 8:**
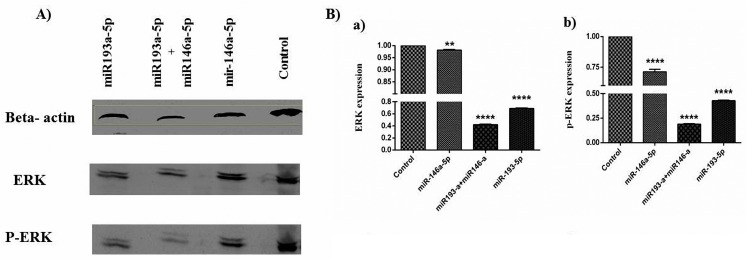
miR193a-5p and -146a-5p controlled the expression of ERK and p-ERK in the CRC cell line. A) The immunoblotting of ERK and p-

To investigate the effects of miR-146a-5p/-193a-5pon the activation of ERK we examined miR-146a-5p and -193a-5p replacement in the HT-29 cells. The replacement of these miRNAsindividually and simultaneouslywas influenced by the activity of ERK, according to results significantly decreased phosphorylation on these proteins. The expressions of ERK and P‐ERK proteins were evaluated by the western blot technique. The miR146a-5p/ -193a-5p modulated the expression of ERK and p‐ERK in the CRC cell line. The miR-146a-5p/-193a-5p individually caused the decrease of ERK, and chiefly the amounts of the phosphorylated protein in comparison to controls, particularly this evidence had a synergistic effect in co-transfected cells (Figure 8).

ERK proteins was assessed in untransfected (control), miR193a-5p, and -146a-5p separately and simultaneously transfected groups. B) (a) ERK

expression, (b) p-ERK expression. **** *P* <  0.0001 versus CTRL cells. ERK: extracellular signal-regulated kinase; miRNA: microRNA; p-ERK:

phosphorylated ERK. The data represent mean ± SD, n = 3, (**P < 0.01; *** *P* < 0.001, **** *P* <  0.0001).

## Discussion

Several studies have described the altered expression of miRNAs in human malignancies. These molecules can target various cellular signaling pathways and modulate the tumorigenesis, angiogenesis, and metastasis of the tumors.^14^ Due to the accumulated evidence about the pivotal role of miRNAs in cancer pathogenesis, nowadays, miRNA-based therapies have been considered as one of the most hotspot issues in the field of cancer therapy.^15,16^ In previous studies, it was reported that both miR-146a-5p/-193a-5p have reduced expression in CRC tissues. Lu et aldesignated the reduced expression of miR-146a-5p in CRC and considered this miRNA as a tumor suppressor gene.^17^ In another study, Zhang et al designated that the expression of miR-193a-5p in colorectal tumor samples was significantly reduced in comparison with healthy samples, and this reduction correlated with the metastasis to the lymph nodes and mortality rate in suffered patients.^18^ Considering these facts, the current investigation was planned to examine the impacts of these miRNAs in colorectal tumor progression either alone or in combination with each other. Here, we showed that miR‐146a‐5p/-193a-5p have decreased expression in metastatic colorectal tumor cells, particularly in the HT-29 cell line. Our data suggest that these miRNAs could represent tumor-suppressive functions, which were in accordance with the earlier studies.^9,19^

The results of western blot analysis displayed the reduced rate of p‐ERK to total ERK in miR‐193a‐5p/-146a-5p transfected cells. These miRNAs synergistically reduced the functions of ERK and caused a decline in cell survival, migratory ability, and proliferation. It is well known that ERK-associated pathways are crucial for cellular actions. Our data was in line with other studies and verified that the expression rate of ERK is at a high level in human CRC tissues.^20,21^ The inhibition of ERK can reduce the survival and migration of the cells. Induction of miR-146a-5p expression individually and particularly in combination with miR‐193a‐5p synergistically reduced the function of ERK and declined cell survival and proliferation. This replacement repressed the activities of ERK and confirmed that the miR‐146a‐5p/-193a-5p have an important role in the RAS–RAF–ERK and related signaling cascades. In this regard, dysregulation of the MAPK pathway in cancers can trigger mutations in BRAF, and KRAS and subsequently, KRAS can motivate Ras– Raf–ERK pathways.^22^ Recent studies revealed the crucial role of the Ras–Raf–ERK in the motivation of tumor cells migration and proliferation.^23,24^ In the current investigation, we established that miR‐193a‐5p and -146a-5p are pivotal factors for the inhibition of both ERK pathways and are essential for the suppression of CRC cell growth, proliferation, and migration. Furthermore, we established that miR‐146a‐5p/-193a-5p might play as an ERK pathway adjuster and a clue to the therapeutic management of cancer-related properties such as proliferation and invasion. The results recommended that as an alternative to targeting the crucial molecules of both the MAPK–ERK pathways, the miR‐146a‐5p/-193a-5p could have beneficial outcomes in treating CRC.

The obtained results from the wound-healing assay also displayed that miR‐146a‐5p/-193a-5p restoration could inhibit the migration of CRC cells and these two miRNAs synergistically suppress the migration of HT-29 cells. This inhibitory effect of miR‐146a‐5p/-193a-5p restoration on the migration of cancer cells may occur through their effects on various genes involved in migration, including CXCR4, E-cadherin, ROCK, and C-Myc. Thus, as the next step of our study, the effects of miR‐146a‐5p/-193a-5p restoration on these genes expression were done by using qRT-PCR. In this regard, Shirafkan et al, and Hurst et al data are concordant with our observations, they displayed that overexpression of miR‐193a‐5p could meaningfully prevent HT‐29 cells migratory ability, and high expression of miR‐146a‐5p could meaningfully suppress MDA-MB-231 cells migration *in vitro*, respectively.^9,25^ The c-Myc transcription factor is one of the downstream components of several kinases signaling pathways, and it is one of the key regulators of cell growth, which has increased expression in various cancers, including CRC, and correlated with increased progression, invasion, and migration of the cells.^26^ According to our results, miR‐146a‐5p/-193a-5p could synergistically decrease the expression of c-Myc. In addition to this, the Rho/Rock/LIMK pathway is crucial in the restructuring of cellular skeletons and cellular migration, and it has high expression in various cancers, including CRC.^27^ Based on our results, this treatment reduced the levels of Rock expression in colon cancer cells. We, furthermore, evaluated the expression of CXCR4 in the HT‐29 cells following miR‐146a‐5p and -193a-5p transfection. CXCR4 is associated with cancer development, invasion, and chemotherapeutic resistance.^28^ We established that the transfection of these miRNAs decreases the mRNA expression ratio of CXCR4 in CRC cell lines. Therefore, this reduction modified the downstream metastasis‐related genes and results in the inhibition of metastasis. Also, the expression of E-cadherin is related to loss of differentiation, tumor progression, metastasis, and invasion in CRC patients.^29^ We demonstrated that our miRNAs mimic transfection reduces the mRNA ratio of E-cadherin in the HT-29 cell line. These observations suggested that miR‐146a‐5p/-193a-5p replacement in CRC synergistically could suppress tumor metastasis. To understand if the miR-146a‐5p/-193a-5p alone or combined could induce apoptosis, two apoptosis assays including FCM and DAPI staining were performed. Apoptosis induction was observed in a miRNA-146a-5p, and miR-146a-5p/-193a-5p transfected groups, and these two miRNAs synergistically induced apoptosis in CRC cells. The study of Zeng et al is in line with our results, as they have also shown that miRNA-146a-5p mimic transfection into the SW-260 cell line inhibited cell growth and induced apoptosis.^30^

## Conclusion

We hypothesized that miR‐146a‐5p and miR‐193a-5p in CRC have a pivotal impact on the proliferation and invasion of cancerous cells. Therefore, by the restoration of these miRNAs, we have examined the effects of them on the cancer cells. The results revealed that miR‐146a‐5p/-193a-5p might be considered as new anti-metastatic and anti-cancer biomarkers in CRC. Furthermore, our findings demonstrated that miR‐146a‐5p/-193a-5p might be novel small therapeutic molecules to repress CRC cell survival, proliferation, and migration through inhibition of the ERK signaling cascade.

## Ethical Issues

All experiments and procedures were conducted in compliance with the ethical principles of Tabriz University of Medical Science, Tabriz, Iran and approved by the regional ethical committee for medical research (Ethical code: IR.TBZMED.VCR.REC.1397.194).

## Conflict of Interests

The authors declare that there are no conflicts of interest.

## Acknowledgments

This work was supported by Immunology Research Center, Tabriz University of Medical Sciences, Tabriz, Iran.
